# The effect of cash transfer programs on educational mobility

**DOI:** 10.1371/journal.pone.0205957

**Published:** 2018-10-19

**Authors:** Florian Chávez-Juárez

**Affiliations:** Laboratorio Nacional de Políticas Públicas (LNPP), Centro de Investigación y Docencia Económicas (CIDE), Mexico City, Mexico; Universidad Veracruzana, MEXICO

## Abstract

In this paper I develop a model to reproduce the phenomenon of high intergenerational correlations in education observed in Latin American Countries. The model is based on empirical evidence and implemented through agent based modeling techniques. The effect of conditional cash transfer programs on educational mobility is then analyzed. The results suggest that conditional cash transfer program can substantially increase intergenerational mobility in education. I find that using parental education as eligibility criterion and adapting the subsidies to the income level can improve the efficiency of a program in increasing educational mobility as compared to a purely income based program.

## 1 Introduction

Following the economic crisis of 2008, economic and social inequalities moved back into the focus of public interest and nourished social movements all over the world. In the U.S. for instance, the *99-percent*-movement pointed to the large and increasing disparities between the very rich people and the main body of the population. [1] argues that not only the state of inequality gives rise to anger, but the fact that social mobility is very low, giving little hope to those at the bottom of the social ladder. Education is probably the key to move up the social ladder as it directly affects life time earnings of individuals ([2–4]). Increasing the number of years of education among the most disadvantaged people in the society is a main goal of many recently introduced conditional cash transfer (CCT) programs. It is therefore interesting to analyze the role of such government programs in promoting educational mobility.

In this study, I develop a model of educational mobility allowing me to analyze the effect of conditional cash transfer programs on the intergenerational link in years of schooling. The model has two main goals. First, I try to reproduce the actual situation in countries with low educational mobility. In a second step, I use the model in order to see whether (conditional) cash transfer programs can increase educational mobility and what properties of these programs matter most. It is important to notice that I focus on the quantity of schooling and assume quality to be constant.

The model is based on a large body of empirical literature, which aimed at measuring the phenomenon and identifying the causes. A complete discussion of the literature can be found in surveys such as [5], [6] and [7]. Among the identified channels of intergenerational transmission of education are the biological transmission of ability ([8–10]) and budget constraints of the parents when investing in the education of their children ([11–13]).

In addition to these two main channels, I also consider several empirical regularities that might be important for educational mobility. Among these regularities, I consider for instance assortative mating and education dependent fertility. Their inclusion in the model can be dissuasive when looking for analytical solutions of a model. For this reason, I make use of techniques from agent based models (ABM), which I consider to be particularly well suited in this context. Agent based models also allow us to easily incorporate and analyze various policy measures without the need of deriving analytical solutions.

The results suggest that conditioning the help on the private education investment of the families and adapting it to the level of poverty makes it more efficient in terms of cost and benefits as opposed to an unconditioned program.

A second result is that the more a government wants to invest, the larger the base of subsidy receiving families should be. Otherwise an overcompensation of the poorest occurs and reduces the effectiveness of the program. In the model a reduction of the intergenerational education correlation from the currently observed level of about 0.55 to about 0.30 can be achieved with a proportional tax rate of approximately 8%. Finally, the model results suggest that using parental education as eligibility criterion and adapting the subsidies to the income level of the family might increase the efficiency of conditional cash transfer programs in terms of reducing intergenerational mobility in education as compared to purely income based criteria.

The paper makes three contributions to the economic literature on educational mobility. First, the model allows me to analyze the potential impact of cash transfer programs on educational mobility. The analysis of different policy schemes aims at identifying the theoretically most efficient ways to reduce intergenerational correlations in education and increase social mobility. Second, by the use of agent based modeling techniques, I can consolidate the theoretical modeling approach and the numerous empirical findings. This should allow me to model intergenerational mobility in education closer to the data. Finally and more generally, the paper should allow me to demonstrate the usefulness of agent based modeling techniques in the literature on social mobility as a complement to the traditional tools.

The remainder of the paper is organized as follows. In section 2, I present some recent literature on educational mobility. Section 3 describes the model with a special focus on how empirical evidence is taken into account. The model description in section 3 is complemented by the ODD protocol in the S1 Appendix and a description of the practical implementation in the S2 Appendix. In section 4 I first present the baseline model to show how it reproduces the status quo and then I analyze the effect of different policy schemes. Section 5 concludes the paper and provides some recommendations and an outlook on possible extensions.

## 2 Educational mobility—Empirical evidence

The aim of this section is to provide a concise and non-exhaustive overview of the literature on educational mobility, focusing on the most important elements for the model I am developing. For a more extensive review of the empirical literature, see [5] or [14] and their references. First, I provide some information on intergenerational correlations in education and then I present some potential driving factors. Before starting this review, it is important to notice that the present study focuses exclusively on the quantity and not the quality of education. Hence, all policy measures will be analyzed in terms of how they increase the number of years of education, implicitly assuming that the quality remains unchanged. Modeling the quality of schooling is beyond the scope of this study.

### Estimation of educational mobility

Education mobility can be measured in various ways including correlations and transition matrices. I use intergenerational correlations because many estimates are available in the literature and a single metric is easier to handle in the theoretical model than matrix based measures.

[15] use the intergenerational correlation of the years of schooling to rank 42 countries. In their ranking Latin American Countries (LAC) depict the highest correlations starting from 0.55 in Nicaragua and going up to 0.66 in Peru. [16] compare only Latin American Countries (LAC) and find that Mexico has the second lowest intergenerational mobility level behind El Salvador. The substantially higher correlations for LAC make them particularly interesting to study. I focus on Mexico to calibrate the model because it is a country with low educational mobility and a good availability of high quality data. [17] also find a correlation of 0.55 for Mexico, while slightly lower values between 0.40 and 0.45 are reported in [14]. However, these last estimates are based on young individuals at the age of tertiary education and represent therefore lower bound estimates for the whole population. Hence, a range between 0.5 and 0.6 seems to be reasonable as a benchmark value for the model that I develop.

### Mechanisms generating low educational mobility

The literature identified mainly three channels of intergenerational transmission: the biological transmission of intelligence and ability, the economic channel and the direct education-to-education channel. I will focus on the former two in the construction of the model and discuss them hereafter in some more details. A completer discussion of the three channels can be found in [14] and its references. In addition to these transmission mechanisms, I also discuss the potential role of assortative mating and education dependent fertility in explaining educational mobility.

#### The biological transmission of ability

The biological transmission channel refers to the genetic transmission of ability. This transmission makes the ability endowments of both generations alike. Assuming that more able people are getting more educated, the years of education between two generations will be highly correlated as a consequence. The inherited part of total ability is substantial according to recent studies. [10] use German panel data and find that a 1 point increase in the IQ score of the parents is associated with a 0.45 to 0.5 point increase in the comparable scores of the children. [8] use Swedish data and find a father-son IQ-correlation of 0.346 and around 0.5 between brothers. Finally, [9] find a father-son IQ-correlation of 0.38 using data from Norway. Using a sample of 7’576 children and young adults from the Mexican Family Life Survey (MxFLS), I find a value of 0.319 for the correlation with the father and 0.366 for the mother, which is in line with the values found for Europe.

#### The economic channel

Under *economic channel* I understand all arguments saying that the economic situation of the family is determined by the education of parents and determines itself the child’s education. This link is due to families’ budget constraints when investing in the education of their children. The problem of budget constraints can be related—but is not limited—to credit constraints. Some authors argue that credit constraints play an important role ([12, 18]). However, an increasing number of authors argue that the long run economic situation of a family, rather than short run credit constraints, is determinant for the economic channel ([11, 14, 19]).

#### Assortative mating

Assortative mating describes the fact that the process of finding a partner is not independent of socioeconomic characteristics such as education. Typically there is a strong correlation in education and cognitive ability between spouses. Therefore, a child will have either a double-benefit from highly educated parents or a double-disadvantage in the opposite case. The analysis of the exact process of partner matching goes beyond the scope of this paperFor more details, see for instance [20] or [21]. [22] and [23] discuss and consider assortative mating in the context of intergenerational transmission of education and argue that it has a potentially important role. [24] use German and British data and find that on average about 40-50% of the covariance between parents and own family permanent income can be attributed to assortative mating. They explain their finding by very strong spouse correlations in education.

#### Education dependent fertility

Education does not only have an effect on the partner finding process, but also affects fertility. Highly educated couples tend to have fewer children than less educated couples ([25]). Particularly the education level of the mother is important in explaining different fertility rates ([26, 27]). Having on average more children among less educated parents directly affects the intergenerational transmission. Less educated parents tend to be poorer and given the larger number of children, they have fewer resources per child to invest in education. Like for assortative mating, I will show in a later section the figures for Mexico that clearly confirm the presence of education dependent fertility.

#### Relevant evidence for the model

The concepts and evidence outlined in this section represent the cornerstones for the model I develop in this paper. The first goal of the paper is to model the mechanism as suggested by the empirical evidence in order to reproduce the intergenerational correlations observed in Latin American Countries. Table 1 summarizes the most important stylized facts that the model aims at reproducing and satisfying.

**Table 1:** Summary of relevant stylized facts.

Description	Value
Intergenerational correlation in education	[0.45–0.65]
Parent-child IQ correlation	[0.31–0.38]
Spouse IQ correlation	≈ 0.40
Spouse education correlation	≈ 0.65
Correlation between education and fertility	< 0

The figures are relatively crude measures of the phenomenon, but they allow me to easily compare the model to the real world. However, to build the model, some more detailed information is needed. In the next section I will introduce the model along with a more detailed discussion of how these empirical regularities are taken into account.

## 3 The model

Based on the empirical evidence and findings of the literature explained in the previous section, I develop in this section a theoretical framework. The aim is first to reproduce the *status quo* and then to analyze different policy measures. The explanation of the model in this section is complemented by the ODD protocol ([28]) provided in the S1 Appendix. The ODD protocol is a standardized way to present agent-based models and aims at increasing their reproducibility. The ODD protocol therefore focuses on the technical implementation of the model, while in this section I discuss essentially the economic reasoning behind the modeling choices.

### General idea of the model

In the model, people live for two periods corresponding to childhood and adulthood. During childhood individuals are getting educated and during adulthood they participate in the labor market and raise their children. Families are composed of two adults and an endogenous number *n*_*c*_ of children. In each period parents decide how much to invest in the education of children and how much to consume. Wages and education are endogenous and defined through equations relating them to ability and family investment in education.

To initiate the model, a set of *N* individuals is created. After the initialization a series of steps takes place in each period. Table 2 displays the four main steps taking place in each period.

**Table 2:** Overview of all steps taking place in each period.

Step	Description
**1**	The adults of the previous period leave the model and former children become adults. They start **searching a partner** and in case of finding one, a new family is created.
**2**	Each family has a positive probability of **getting children**, where the number of children depends on the education level of the mother.
**3**	Each **family earns a salary** and pays taxes.
**4**	Based on all private information of the family, the parents decide how much to **invest in the education of children** and how much to consume. Children receive education according to the investment.

Once these steps are completed, the next period begins and the sequence starts again. Hence, in every simulation we will have several consecutive generations and we will be able to analyze how a policy measure affects these generations over time. I will now present each of these steps in more detail and highlight how and to what extent empirical evidence was used in the modeling process. Additionally, an overview of all parameters in the baseline model can be found in the S2 Appendix.

### Step 1: Partner search with assortative mating

Modeling the partner matching process in accordance with the empirical evidence on assortative mating can be done in several ways. For this model, I chose a very simple solution, where individuals with too large differences in terms of education and ability cannot form a couple. The threshold was defined by consulting the Mexican Life Family Survey, which shows that for 95% of the couples the IQ difference does not exceed 30 points and in the case of education 95% of all partners are within 1.5 standard deviations. The two conditions are overlapping for a relatively large part of the population. However, using only one of them yields less satisfactory results than using them together. In the model, potential candidates for a partner must therefore satisfy the following conditions:

$$\begin{array}{cc} \delta_{IQ}\equiv \left|IQ_{woman}-IQ_{man}\right|\leq 30 & \;\wedge \;\delta_{educ}\equiv \left|educ_{woman}-educ_{man}\right|\leq 1.5\sigma_{educ} \end{array}$$ (1)

This double condition is a very simple way to account for assortative mating. Much more sophisticated algorithms could be imagined. However, as I will show in the result section, this simple implementation produces results that are very close to the spouse IQ-correlation of 0.400 and the spouse education correlation of 0.64 reported in [14].

### Step 2: Procreation

Once couples are formed, they have a positive probability of getting children, each of them having an endogenously defined level of ability. Let us first define the number of children and then their ability level.

#### Education dependent fertility

**Fig 1:**
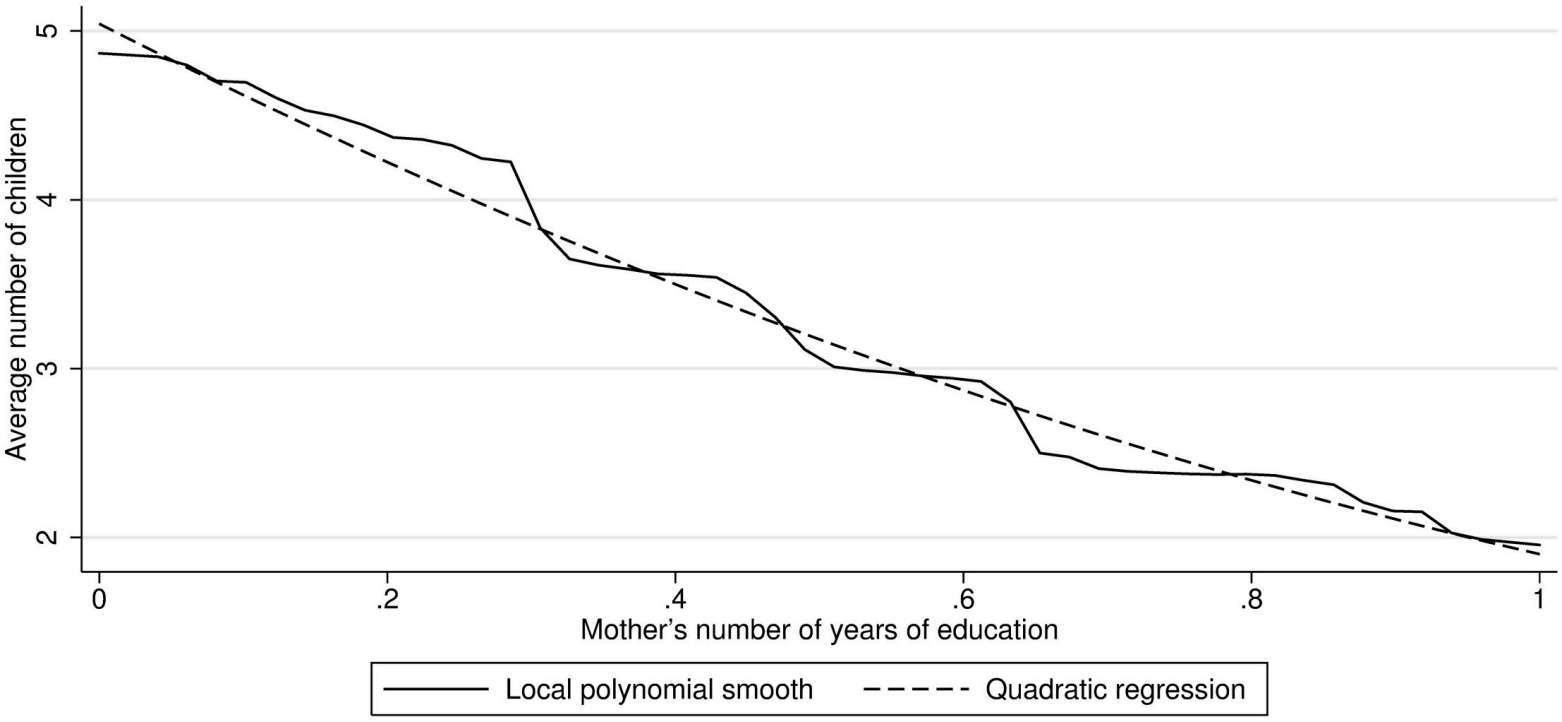
Number of children as function of maternal education. *Source*: Based on data from the Mexican Census 2010.

The expected number of children depends on the education level of the mother. To model this phenomenon, I use Mexican data and implement the link between mother’s education and the number of children accordingly. Fig 1 displays the Nadaraya-Watson estimator ([29, 30]) and the quadratic OLS fit of the number of children in function of the education of the mother based on data from the 2010 Mexican Census. For simplicity, education is normalized to the interval of 0 (no education) and 1 (highest education in the population).

In the model I use the conditional expectation obtained from the parametric fit as the argument of a Poisson distribution. This allows me to reproduce a certain level of heterogeneity in the number of children for a given level of education. Thus, the actual number of children a couple has is given by

$$\begin{array}{c} n_{c}\left(e_{m}\right)∼P\left(E\left[n_{c}|e_{m}\right]\right) \end{array}$$ (2)

where *E*[*n*_*c*_|*e*_*m*_] is the expected number of children conditioned on the level of education of the mother using the above mentioned parametric model. In the ODD protocol I present the detailed estimates of the parameters and an explanation of the sample.

It is worth noticing that in this model the number of children is not the result of the optimization problem of the family, as this does not seem to be appropriate for the Mexican case. Moreover, the focus of this paper lies on possible policy measures, thus families would have to anticipate the subsidies before getting children. This is beyond the scope of this paper.

#### Ability transmission through genes

Besides the endogenous number of children, their ability level is also endogenous in the model and directly depends on the ability level of the parents. To approach as much as possible the actual transmission of cognitive ability, I make use of the Mexican Family Life Survey (MxFLS), where a short cognitive ability test is included for both parents and their children. By regressing the child’s IQ on the IQs of both parents we obtain the following equation:

$$\begin{array}{c} a_{\text{child}}=51.8772+0.2059\cdot a_{\text{father}}+0.2857\cdot a_{\text{mother}}+N\left(0,13.53\right) \end{array}$$ (3)

where the IQ of the mother (*a*_mother_) matters significantly (F = 17.05) more than the one of the father. The regression also allows us to estimate the distribution of the random part. In the ODD protocol in the S1 Appendix I provide more detailed information on this estimation.

### Step 3: Earning a salary: The wage equation

The wage *w* of each adult depends on his or her education *e*. The link between education and wages is generally analyzed using the log-linear specification as proposed by [31]. I follow this approach and obtain the following exponential expression of the wage level:

$$w=\text{exp}(\beta_{0}+\beta_{1}e+\epsilon_{w})$$ (4)

where the *β*’s are parameters and *ϵ*_*w*_ is a random disturbance term accounting for unobserved heterogeneity in the agents. In order to simplify the implementation in the model and to have a currency independent equation, I normalize the income of individuals without education (base income) to 1. This normalization shapes the interpretation of *w* to a multiple of the base wage obtained without education and implies that *β*_0_ = 0. Education is also normalized to the interval between zero (no education) and one (highest possible education). The parameter *β*_1_ is then estimated using the 2010 Mexican Census. For the analysis, very few top-coded wages were excluded. The model includes 2’085’318 observations and has an *R*^2^ of 0.603. The estimated parameter is 1.607, which corresponds to a return to one year of education of about 9%. The estimated error term has a standard deviation of 0.756 and this value is directly used to simulate *ϵ*_*w*_. Thus, the calibrated wage equation is given by:

w=exp(1.607e+N(0,0.756)) (5)

I present sensitivity checks of these parameters in the S3 Appendix. Note that this implementation implicitly assumes that the quality of education remains unchanged, i.e. that a given number of years of education is comparable across time and socio-economic groups. As a consequence, all results shall be interpreted as *ceteris paribus* effects of conditional cash transfer programs on the intergenerational link in years of education.

### Step 4: Investment in education of children

The education investment decision is the crucial decision taken by families in each period. The education production function is assumed to be common knowledge. The family faces a trade-off between current consumption and investment in the education of children. First, I introduce the education production function and afterwards I come back to the optimization process. Education depends on the investment *i* in education and the ability level *a* of the child. I assume the general form of the education production function:

$$\begin{array}{c} e\left(a,i\right)=\gamma_{1}i^{\gamma_{2}}a^{\gamma_{3}}\epsilon_{e} \end{array}$$ (6)

where the *γ*’s are parameters and *ϵ*_*e*_ is a random disturbance term to account for non-observed heterogeneity. *γ*_2_ is the parameter shaping investment to education. The proposed functional form ensures that investment in education and ability are imperfect substitutes. A very smart child who receives absolutely no support for education is likely to drop out of the schooling system quite quickly. On the other hand, even with massive investments in education, a child with a very low level of intelligence will not completely succeed in school. The fact that education will be zero when families do not invest at all might be a surprising assumption. I justify this choice by the definition of education and investment in this case. First, education can be seen as formal education that adds to some basic knowledge children would acquire in any case. By looking at the wage equation we can see that without education there is still a positive wage. Second, investment should be seen in the larger sense and not be limited to paying school fees. In this respect, even very poor families can invest in the education, e.g. by the simple fact of sending their children to school instead of letting them work at home.

The education production function is indirectly calibrated. The method of this indirect calibration will be explained at the end of this section.

#### The optimization problem of the family

Knowing the education production function, the family has to optimize the investment in education taking into account that it reduces resources for current consumption. We can express total consumption as:

$$\begin{array}{c} C=W-I-T\left(W\right)+S\left(i,n_{c},W\right) \end{array}$$ (7)

where the capital letters *C*, *W* and *I* refer to the family level of consumption, wages and investment respectively. *T*(*W*) is the amount of taxes the family has to pay in function of their labor income and *S*(*i*, *n*_*c*_, *W*) is the amount of subsidies the family receives from the government. In the absence of a government, the last two terms are simply equal to zero.

Families derive utility from their current consumption level and the expected education level of the children. An alternative way would be that families derive utility from the expected future earnings of children. Since future earnings are monotonically increasing in education, it will give the same results, once the functional form of the utility function is adapted coherently. I assume intergenerational altruism given that investment in education has no direct return for the older generation. We can therefore assume for instance the following form of the utility function:

$$\begin{array}{c} U\left(C,E\left[e_{c}\right]\right)=C^{\alpha }\prod_{i=1}^{n_{c}}E{\left[e_{c}\right]}^{1-\alpha }=C^{\alpha }{\left(E{\left[e_{c}\right]}^{n_{c}}\right)}^{1-\alpha } \end{array}$$ (8)

where *E*[*e*_*c*_] is the expected education level of the children and *C* the current consumption level, both a function of investment in education. This formulation ensures that the total investment in education increases with the number of children, while the investment per child is decreasing.

As current consumption and expected education are determined by the investment in education per child *i*, we can express the utility as a function of *i*.

$$\begin{array}{c} U\left(i\right)={\left[W-i\times n_{c}-T\left(W\right)+S\left(i,n_{c},W\right)\right]}^{\alpha }{\left[E{\left[e_{c}\left(a,i\right)\right]}^{n_{c}}\right]}^{1-\alpha } \end{array}$$ (9)

The family then maximizes this utility function with respect to *i*, where the solution depends on the number of children, the income of the family and the form of *T*(*W*) and *S*(*i*, *n*_*c*_, *W*).

### Calibration of non-estimated parameters

Most parameters shown before were calibrated through econometric estimations with Mexican data. However, there are a few parameters for which the empirical estimation is not possible. This concerns particularly the parameters of the education production function (Eq 6) and the parameter *α* in the utility function. Instead of simply assuming some plausible values, I performed an indirect calibration of the model. The idea is to consider several key statistics observed in the data and to simulate the model for different sets of parameters. I consider the intergenerational correlations in education with both the father and the mother and additionally three indicators of the education distribution. Besides the first two moments of the education distribution, I also consider the proportion of individuals with very high education (at least 0.8 on the scale from 0 to 1).

I then chose the parameter set that minimizes the average mean squared error of all five indicators as criterion. I use equal weights for all indicators when computing the average mean squared error. Changing the weights does not alter the conclusion on the best set of parameters. The set of parameters with the smallest mean squared error was chosen for the baseline model. In order to analyze the impact of this choice on the results, I present sensitivity checks of these parameters in the S3 Appendix.

### Scope of the model

Social mobility is a very complex phenomenon and it is important to bear in mind that this model focuses only on the quantity effect of government programs. The idea of this effect is that—*ceteris paribus*—less intergenerational persistence in the quantity of schooling will positively affect social mobility. If the government program also affects the quality of schooling, the results produced by this partial model could be over- or under-estimate the true effects. For instance, if the additional education achieved by children in low socioeconomic groups is of lower quality than the education for the better off, then the beneficial effects of the model might be exaggerated. At the same time, if the conditional cash transfer program also includes measures to increase or improve the school supply, the true effect might even be larger than what the model produced. Including such quality aspects to the model is beyond the scope of the study.

## 4 Results

The results of the model are reported in two steps: first I discuss the baseline model without any policy intervention and then I move on to the analysis of different policy measures. The goal of the baseline model is to show how the model fits the empirical evidence and how the different components of the model contribute to it. The section on policy interventions represents the active use of the model to predict hypothetical responses of the model to different policy measures. These policy measures are inspired by actual conditional cash transfer projects, such as *Oportunidades* in Mexico or *Bolsa Familia* in Brazil. In addition to these results, I present several sensitivity checks on the baseline model and the policy intervention analysis in the S3 Appendix.

### Baseline model and sequential implementation

The primary goal of presenting the baseline model without policy intervention is to highlight the relative importance of the different transmission channels. For this purpose I present the full model along with reduced models where I exclude several elements. By comparing the generated intergenerational correlations of the full model and the reduced models we can see how important the different elements are.

Table 3 summarizes the main statistics of the full model and the relative importance of the different elements. The different statistics of the model are displayed in columns, while rows refer to different settings by including and excluding some elements.

**Table 3:** Average statistics of full model and loss when excluding components.

		Edu. corr. partner	Edu. corr. mother	Edu. corr. father	IQ corr. father	IQ corr. mother	IQ corr. partner
		(1)	(2)	(3)	(4)	(5)	(6)
	**Full model**						
[1]	Correlation	0.719	**0.533**	**0.490**	0.324	0.370	0.441
	Standard deviation	(0.016)	**(0.037)**	**(0.041)**	(0.025)	(0.021)	(0.033)
	**Excluded element**:						
[2]	Assortative mating	−99.8% **	−25.9% **	−40.3% **	−37.0% **	−22.9% **	−100.8% **
[3]	Biological ability transmission	−0.7% **	−5.0% **	−5.4% **	−100.2% **	−100.7% **	n.s.
[4]	Educ. dependent fertility	−0.9% **	−10.9% **	−3.1% **	n.s.	n.s.	n.s.
[5]	Combination of [2] and [3]	−100.1% **	−29.2% **	−43.3% **	−100.3% **	−99.7% **	−99.6% **
[6]	Stochastic wages	−1.2% **	27.9% **	28.1% **	n.s.	n.s.	2.1% **
[7]	Stochastic education	−0.5% **	3.7% **	4.0% **	n.s.	n.s.	n.s.

**Notes**: The first panel called ‘Full model’ displays the average values of the statistics in the full model. The second panel shows the percentage change in these statistics when excluding the element indicated in the first column. Only significant losses are reported. Significance levels: * = 5%, ** = 1%, n.s. = not significant at 5%. Each statistic is the average of 400 data points stemming from 40 steady-state periods and 10 different random seeds.

The first panel of values (row [1]) refers to the baseline model, where all elements are activated. In the first three columns the education correlations are displayed, starting with the spouse correlation, followed by the correlation of the child with the mother and the father respectively (column 2 and 3). The spouse education correlation of 0.719 is slightly higher than in the data and results from the partner eligibility rules discussed in section 3. The education correlation of the child with the parents is the fruit of all processes involved in the full model and also attains values close to the observed figures. The correlation with the mother is somewhat higher than the correlation with the father. This corresponds to the differences found in the data.

The IQ correlations between the different agents are directly based on the calibration. First, the correlation between spouses reported in column (6) follows from the assortative mating restriction (Eq 1). The IQ correlations of the child with the parents are resulting from Eq (3) and reproduce the empirical facts very well.

The second and larger panel in Table 3 reports how these figures change when the element indicated in the first column is excluded. Note that only changes that are significant at the 5% level are reported.

Row [2] displays the generated correlations when assortative mating is not considered in the model, thus when all individuals are potential partners. By construction, the spouse correlations go to zero, since the matching process becomes completely random. More importantly, we can observe a sharp decrease in the intergenerational correlations in education and ability. The decrease is particularly important for the correlation with the father, but even in the case of the mother the correlation falls by more than 20%. This finding is due to *regression to the mean* and illustrates that the exclusion of assortative mating would be very problematic.

Row [3] shows what would happen if the transmission of ability trough genes would be excluded from the model. In this case the intergenerational IQ correlations are by definition driven to zero, since ability becomes a purely random characteristic. The intergenerational education correlations are also reduced by slightly more than 5% of their initial values. The spouse education correlation is significantly affected, but the change remains very small and economically rather irrelevant.

In row [4], the correlations of the model when excluding the education dependent fertility rate feature are reported. The reduction is much higher for the mother than for the father. Comparing this to the correlations of the full model, we can see that when ignoring this empirical regularity, the simulated correlations for both parents are much closer to each other. This would be in contradiction to the data, which suggests that the correlation with the mother is higher. Hence, even though the changes are not very high, the inclusion might be justified by the difference in correlation with each of the parents.

Row [5] presents the figures when the assortative mating and the biological transmission of ability are simultaneously excluded. The reduction in the education correlations is slightly less than the sum of the two individual exclusions. Nevertheless, there does not seem to be a major interaction effect between the two elements.

Excluding the stochastic term in the wage equation has a large and positive effect for the intergenerational correlations in education. Due to the non-linearity of the log-transformation, the expected wages without stochastic term are lower than they would be with the stochastic term. For this reason, I use the smearing factor proposed by [32] and illustrated by [33] to keep the same average income level and to avoid confounding the effect of excluding the stochastic term with a change in average income. The reason behind this result is that with the stochastic elements even poorly educated parents can be lucky and earn more than the expected income given their education. In such a situation, they will be able to invest more in the education of the children. In absence of these stochastic deviations from the mean, people with low education will always have small wages and therefore they will not be able to invest more in the education of their children.

In contrast, the exclusion of the stochastic term in education does not seem to have a major effect. It also increases slightly the intergenerational correlations, but by a rather small amount.

Overall, the baseline model succeeds at reproducing the main stylized facts appropriately and the exclusion of some elements of the model substantially affects the main statistics in some cases. Nevertheless, it would be possible to reproduce the stylized facts without these elements by the mean of a re-calibration. Such an approach would, however, suffer from the fact that some empirical evidence is left aside. Such a reduced model would—as a consequence—put too much weight on the core processes of the model, especially on the optimization process. As a result of that overweighting, the effect of policy interventions is likely to be overestimated too. For this reason, all additional features such as assortative mating or the transmission of ability through genes are kept in the model in the following section where I introduce the government.

### Policy measures

Based on the baseline model just described, I now present how the key statistics react to the introduction of different policy schemes. All measures I am introducing should be understood as additional measures compared to the *status quo*. All results are based on comparisons of steady states. Steady states refer to the period when the model is stable after initialization and again stable after the policy intervention starts. In the S2 Appendix I explain in detail how the simulations were performed and what periods were considered for the results. It also provides an overview of the used data for each Figure and Table in this section.

The general idea of the policy measures is to give monetary subsidies to families allowing them to invest more in the education of their children. All subsidies are paid by a proportional tax. In a preliminary study I tested various conditional and unconditional policies and found out that double conditioned measures always provide more efficient results (for details, see [34, Chapter 3]). For this reason, I focus here exclusively on double conditioned programs. The double conditionality refers to conditioning subsidy on private investment in education and on the deprivation level of the family. In this sense, families receive more subsidies when they invest more on their own in the education of their children and poorer families receive larger subsidies than relatively less poor.

Traditional (conditional) cash transfer programs essentially focus on economic variables to determine the eligibility of families. However, in our context it might also be interesting to see whether using parental education as eligibility criterion and to determine the amount of subsidies could yield more interesting results. Table 4 displays the resulting four possible combinations of policy measures.

**Table 4:** Double conditioned and mixed schemes.

Determinant for subsidy	Eligibility criterion		Subsidy level
	Income	Education	
Income	II	EI	$S=(1-\frac{W}{\omega })\eta in$
Education	IE	EE	$S=(1-\frac{e_{p}}{\omega_{e}})\eta in$
Eligible if	*W* < *ω*	*e*_*p*_ < *ω*_*e*_	

**Note**: *Income* refers to family income and *Education* to the highest parental education level. *i* refers to the private investment per child of the family, *n* is the number of children and *η* is a parameter for the size of the program.

We can use the family income or the highest parental education for both the eligibility criterion and the calculation of the subsidy, but also a mix of the two indicators. For instance, we can base the eligibility on parental education and then determine the amount of the subsidy based on the income of the family. The eligibility criterion is simply a threshold dividing the population in eligible and non-eligible families. The amount of the subsidy depends on the distance to the threshold $\left(1-\frac{W}{\omega }\right)$ and $\left(1-\frac{e_{p}}{\omega_{e}}\right)$ respectively, the amount of private investment *i*, the number of children *n* and a parameter *η*.

It could be argued that conditioning subsidies on private investment is not suitable in the context of very poor families as they might not be able to invest at all. This is true when considering private investment in the narrow financial sense. However, I argue that the private investment should be seen as a broader concept. For instance, school assistance instead of child labor is a sort of investment in terms of opportunity cost. It is important to mention that subsidies are always positive or zero. Thus, the only payment to the government goes through the taxes. For all schemes, the corresponding tax scheme is a proportional tax on the sum of all wages of the family. In order to ensure that the amount of taxes corresponds to the amount of subsidies, the tax rate is adapted endogenously. The tax rate in period *t* is set in order to cover the expenses on subsidies of period *t-1*. As a consequence, each generation pays as adult for the subsidies received as child, making each generation paying for their own subsidies. It could be argued that this is problematic for the first period because no taxes have to be paid. However, the results in this study do not depend on this because I exclude the first periods from the analysis (see S2 Appendix for more details). This post-paid implementation is justified by the endogenous nature of the program size. The total amount of subsidies depends on the decision made by families, which depends itself on the amount of taxes. By paying the subsidies of the previous period, we can fix the amount of taxes and subsequently families can optimize their investment in education. This way, the model ensures that all subsidies are paid by taxes and the government does not incur debt for the financing. This implicitly assumes that the government can borrow money at no cost, otherwise the debt in *t* would be higher that the subsidies paid in *t* − 1. To deal with this, I ran additional simulations where I introduced an interest rate for the government debt. Introducing this interest rate makes the policy interventions somewhat more expensive, but does not at all change the comparison of the different schemes. Hence, for the sake of reducing the amount of parameters, I present the results without interest rates.

**Fig 2:**
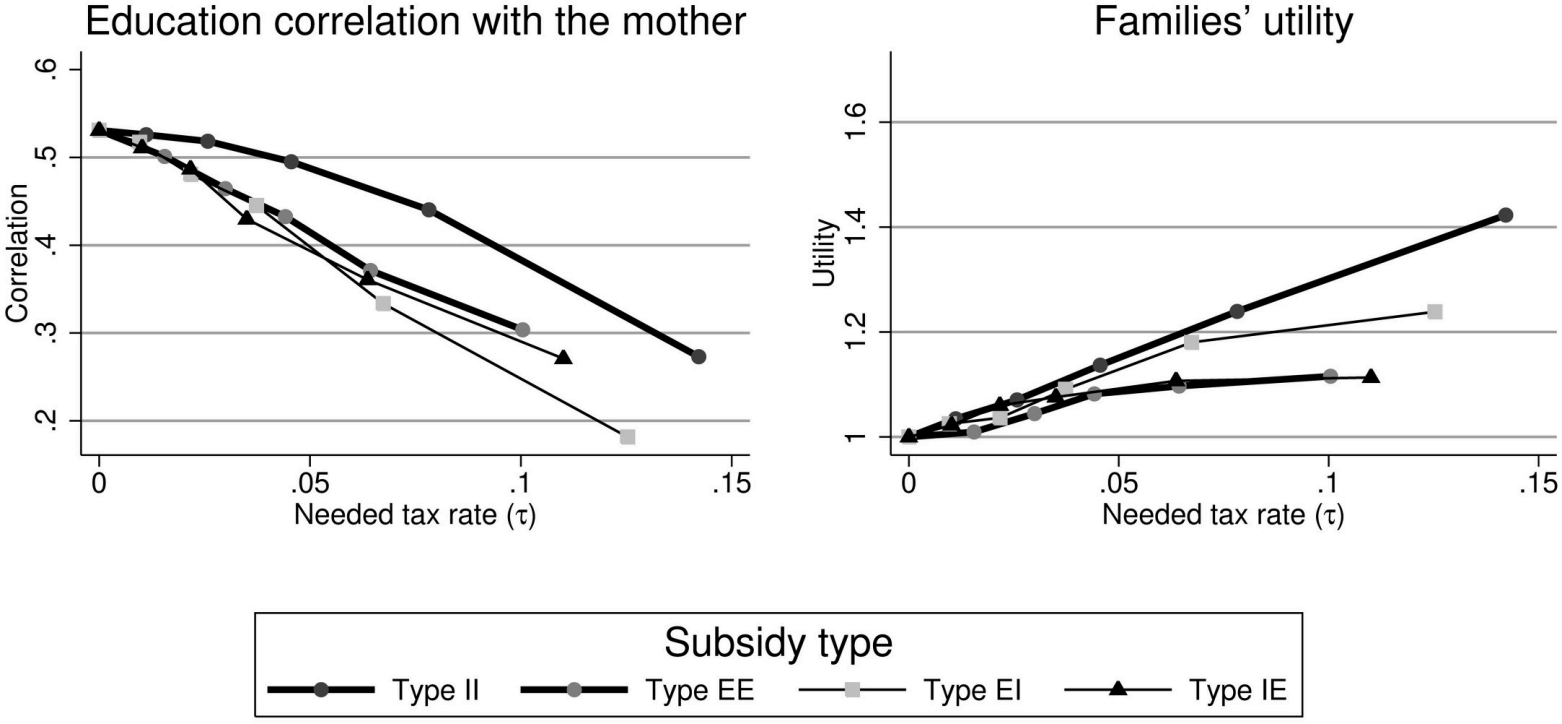
Comparison of different subsidy schemes. **Notes**: Each point in the graphics is based on 80 population statistics. These 80 statistics stem from 20 different steady-state periods from 4 different random seeds.

Fig 2 displays the effects of the different policy schemes on the intergenerational correlation with the mother (left, almost identical for the father) an on the average utility of families (right) in function of the needed tax rate. Each point in the graph corresponds to a specific setting of the simulation and refers to the average value over periods and simulations. In the S2 Appendix I present all the technical details and the considered values for each of these simulations. The points are vertically not on the same values because the changing parameter when moving to the right is *η*. The reported *θ* on the horizontal axis is endogenously determined.

First and most importantly all types of policy measures significantly reduce the intergenerational correlations in education and increase the average utility level. Among the four analyzed schemes all but type II reduce the correlations similarly. For a given tax level type II reduces the correlations less and is therefore less efficient in terms of increasing educational mobility. In contrast, when looking at the increase in utility we can observe that the type II scheme performs best, followed by the EI scheme. The EI scheme is therefore the most promising approach as it performs well in both decreasing the correlations and increasing the average utility. The intuition behind the different rankings is the following. By subsidizing those parents with little education and therefore by increasing the expected education of their children we directly address the intergenerational link in education. In contrast, focusing exclusively on the income dimension increases the average utility more because of the decreasing marginal utility return of income. The fact that EI performs better than EE in terms of increasing utility is due to the fact that the marginal utility of income is highest among the poorest and therefore the subsidy is more efficient. Hence, combining both parental education and their income level to determine who receives how much subsidy is performing best because both dimensions are taken into account.

I will now start discussing the effects of these programs and the role of some policy relevant parameters like the eligibility threshold in more detail. For this more detailed discussion, I will focus on the mixed strategy (Type EI), where parental education is used as eligibility criterion and the subsidy level is based on the income level of the families.

#### A closer look at the link between parental and offspring’s education

To simplify the discussion, let us focus on only a limited number of cases and only Type EI interventions. I use the value of 0.5 as threshold and I limit the analysis to only three levels of intervention which I denominate small, moderate and strong. The small intervention incurs a tax rate of 4%, the moderate of 7% and the strong intervention requires a tax rate of approximately 13%.

**Fig 3:**
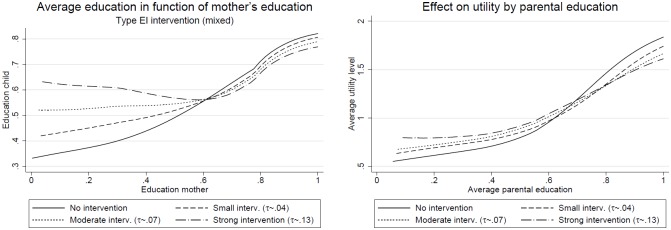
Effect of the subsidy (Type EI) on the education correlation with the parents. **Notes**: Each curve is based on approximately 210,000 individuals. These individuals were obtained from 21 periods for 10 different random seeds.

Fig 3 shows the average education of children (left) and the average utility level (right) in function of the parental education using the non-parametric kernel regression. I use the kernel regression of degree 0, which is commonly known as the Nadayara-Watson estimator of the conditional mean ([29, 30]). This allows us to better understand the effects of the program.

Let us first consider the average education level of the child displayed on the left graph. In the case of no state intervention, we observe an increasing and slightly S-shaped function, starting at values of 0.35 and going up to 0.8 approximately. For the small and moderate intervention the curve turns around the midpoint, making the children of very little educated parents better off, while those of well-educated slightly lose. The increase for the children of parents with little education is due to a net transfer to them, while the reduction at the upper tail of the distribution is due to the income tax and therefore to fewer resources. Note that for the moderate intervention (dotted line) the level of education is stable for mother’s education below 0.45 approximately, while we can observe an overcompensation of the children with the least educated mothers under the strong intervention.

Two things should be learned from this case. First, it is easy to see that the group of eligible people should be enlarged in the case of a large intervention. This would allow the children of parents with a middle high education to benefit as well. Second, the beneficial situation for the least favored children is always accompanied by a decrease in average education for the best-off students, which is straightforward from the fact that the policy measure has a redistributive character.

Let us now consider the right hand size graph, where we observe the average utility in function of parental education. Here I use the average parental education on the horizontal axis rather than only mother’s education. Alternatively we could produce the same graph for both the father and the mother individually. However, they look very much like the one based on average parental education.

Similar to Fig 3 we can see an S-shaped function of the utility level. When introducing the subsidy schemes, the average utility increases at the bottom and decreases at the top of the parental education distribution. The intuition is similar as before. At the lower tail of the parental education distribution people receive subsidies and use them to increase their utility either by investing more in the education of their children or by consuming more. On the top of the distribution less money is available and therefore the utility level is slightly reduced.

Hence, for both the education of the children and the utility level the differences between the best- and worst-off are reduced. Let us have a look at the average levels of both education and utility to see whether the losses at the top are compensated for by the gains at the bottom.

#### Effects on average education and consumption

**Fig 4:**
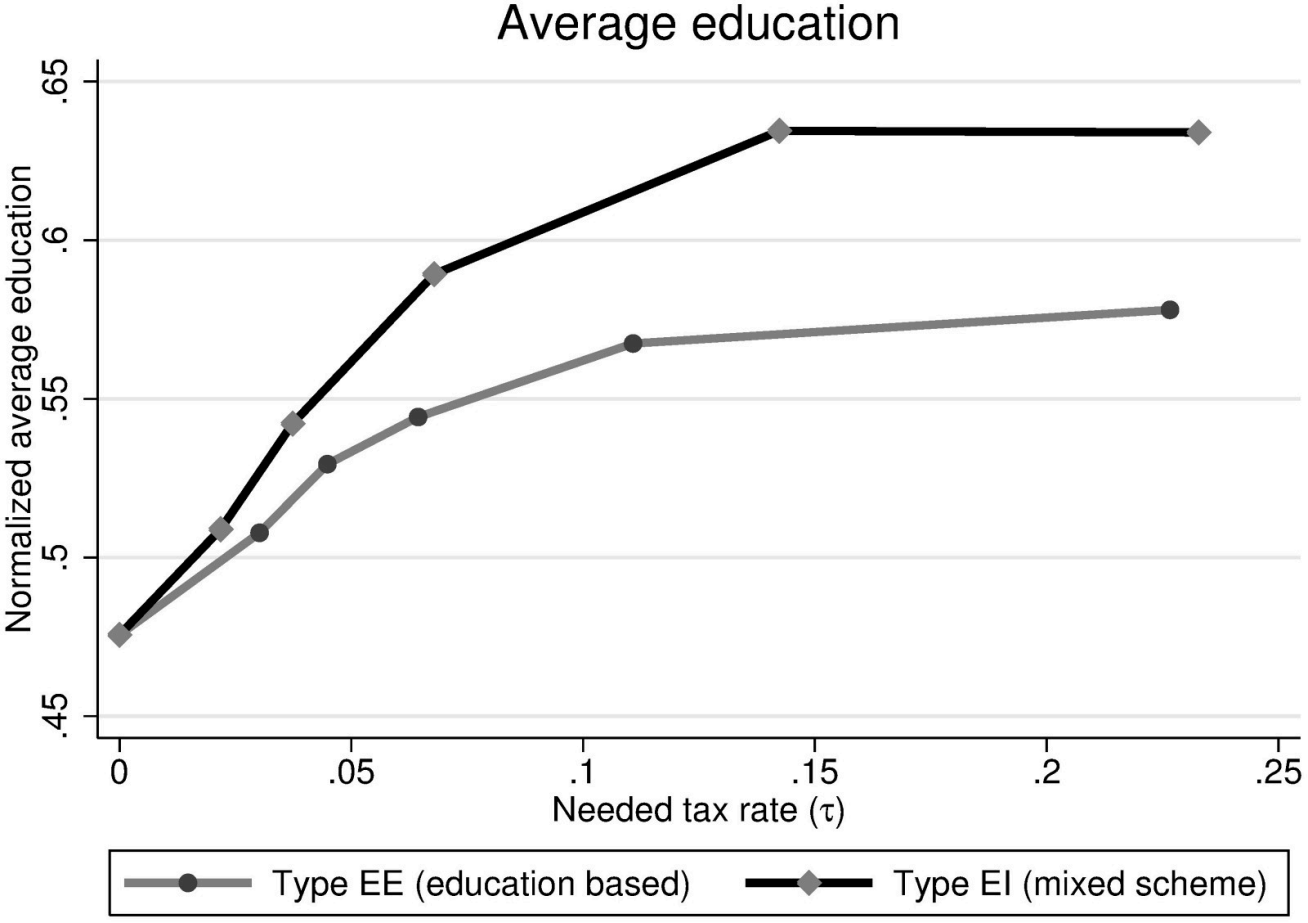
Effect of the subsidy on the average education. **Notes**: Each point in the curves is based on 100 steady-state population statistics. These were obtained from 20 periods for 5 different random seeds.

Let me first discuss the increase in average education by looking at the relationship between the increase and the size of the government program. Fig 4 shows the average education in function of the tax rate used to finance the subsidies for both types of interventions.

The relationship between average education and the tax rate is clearly positive and concave, suggesting that the negative effects for the net payers are more than offset by the positive effect of the net receivers. The increase in average education is larger when the income level determines the amount of subsidies (Type EI) as compared to parental education as determinant (Type EE). The intuition behind this result is that the marginal effect of additional money on education is larger for the poorest individuals. By giving more money to the poorest, this money produces more additional years of education than if we would give it to somewhat richer families.

## 5 Conclusions

In this study I develop a model to reproduce the high intergenerational correlations in education observed in Latin America. I consider two main generating processes: the biological transmission of ability and economic constraints for the investment in education. The use of agent based modeling techniques allows me to take into account several empirical regularities like the education dependent fertility and assortative mating.

I use data from Mexico to calibrate the model, which allows me to reproduce several key statistics as they are observed in the data for Latin American Countries. Among the reproduced statistics are the intergenerational correlations in education and ability and the distributions of education and income. Assortative mating seems to matter substantially, as the intergenerational education correlations drop sharply when not including it in the model. When excluding the education dependent fertility the correlations also drop below the values observed in the data and contrary to the data the correlations with the father and the mother become very similar. It is therefore required to consider these empirical regularities in such a model.

The model is then used to simulate the effect of different policy schemes on educational mobility. Different subsidy schemes, all paid by a proportional tax, are simulated and their impact on educational mobility compared.

Several policy relevant lessons can be drawn from this simulation exercise. First, subsidies to families with children are likely to increase educational mobility and to have positive effects on the average education level. The gains for subsidy receiver more than offset the losses for the net contributors at the top of the income or parental education distributions. This finding holds for all policy schemes.

Second, the eligibility threshold based on the highest parental education yields higher reductions in intergenerational correlations in education as compared to income as criterion. Therefore, using parental education and parental income to define who receives how much subsidies is likely to make programs more efficient. However, it could be argued that it would be easy for families to underreport the educational attainment. While this is true, it also applies to income or to a certain extent to family assets. The advantage of education as criterion is that there is no learning about how the program selects households. Therefore, households will not be able to hide the assets that matter more for the reevaluation. In practice, a combination of several indicators is most likely to be used and therefore the argument might simply be to give some more weight on parental education.

Third, the share of people eligible to receive subsidies should change in function of the size of the program. Larger programs should have a higher eligibility threshold than smaller programs.

However, it is important to mention two caveats of the present study. First, one might want to consider the introduction of poverty sensitive subsidies in conditional cash transfer programs instead of giving the same amount to all beneficiaries. Such a differentiated subsidy scheme is likely to make the program more efficient. However, they might also introduce additional administrative costs and jealousy among receivers, which could in turn reduce the efficiency gains. The inclusion of administrative cost is beyond the scope of this analysis. However, it should be noted that the ranking of the different schemes could change if the administrative costs vary a lot between them. Second, the present study focuses exclusively on the quantity of education and assumes that everything else remains constant. In particular, the model assumes that the additional years of education are of the same quality, which might not be true for a variety of reasons. For instance, the quality of schools and teachers might be lower in poor regions and therefore the gains from the additional years of education would be lower than what is assumed in the model. Analysis such quality effects is beyond the scope of this study, but it might be an interesting and important topic for future research.

More generally, it is important to notice that this study is only a first step in modeling educational mobility with ABM techniques. In addition to the aforementioned caveats, I see mainly two ways for future research. It could be very interesting to complement this model of overlapping generations by a model that focuses more on the short run dynamics. The current model does not enable us to study such effect because the time unit is a generation. A second direction of future development could include the inclusion of even more pieces of empirical evidence and the calibration of the model to a very specific context, e.g. an existing social assistance program.

## Supporting information

S1 Appendix ODD: Overview, design concepts, and details protocol. The ODD protocol is a standardized protocol for the presentation of agent-based models.
(PDF)

S2 Appendix Overview of simulations. This appendix provides an overview of the different simulations and how the data were obtained to generate the results presented in the study.
(PDF)

S3 Appendix Sensitivity checks. This appendix provides a series of sensitivity checks of key parameters of the model.
(PDF)
