# Supplementary material for: The effect of cash transfer programs on educational mobility
Source: PLoS One. 2018 Oct 19;13(10):e0205957. doi: 10.1371/journal.pone.0205957 (PMC6195296; doi:10.1371/journal.pone.0205957)
Supplement: S1 Appendix — The ODD protocol is a standardized protocol for the presentation of agent-based models. (PDF) [file pone.0205957.s001.pdf]

# S1 ODD: Overview, Design concepts, and Details protocol

In addition to the model description in section 3 of the article, I present in this appendix the Overview, Design concepts, and Details (ODD) protocol of the model (Grimm et al., 2006, 2010). The ODD protocol became the standard way of presenting agent-based models. The goal of this protocol is to provide in a concise and structured way all important elements of the model. This should allow other researchers to reproduce the model. In contrast, in this protocol I do not discuss all the justifications of the model choices, as this is done in the main part of the paper, particularly in section 3.

## S1.1 Overview

### S1.1.1 Purpose

The purpose of the model developed in this study is twofold. First, it aims at reproducing the high intergenerational correlations in education observed in Latin America. Second, the model is used to analyze the effect of cash transfer programs on intergenerational correlations in education and the utility level of families. The model is implemented as agent based model allowing me to include several empirical regularities, such as assortative mating and education dependent fertility.

### S1.1.2 State variables and scales

The model is based on a set of three nested entities. The top level entity is the **government**, which is implemented in a very simple and not explicit way. The second level is the **family**, which is composed itself of **individuals**. I will now discuss each of the entities by starting at the lowest level, the individuals and then moving up.

***Individuals***
Table S1.1 describes the different state variables of individuals. Individuals' age can take only two values because the time unit in the model is a generation. Hence, individuals live for two periods only: childhood and adulthood. Education is a continuous variable on the interval from zero (no education) to one (highest possible education). I use a continuous variable here because I do not model the different education levels (primary, secondary, etc.) explicitly. Adding the levels of education would make the model more complicated and does not seem to be very relevant in this long run model[1]. Finally, the wage level of the individual is based on a continuous variable, which I normalized to 1 for individuals without education. This normalization is particularly helpful in the calibration exercise where I use empirical data from Mexico. Moreover, the variable

[1] In a short run model where siblings are not all at the same education level at the same time this might become important. For instance, families could give priority to the child that is close to completing a level. However, this is beyond the scope of the model presented in this study.

Table S1.1: State variables of individuals

| **Variable** | **Description** | **Scale** |
|---|---|---|
| Age | Age describes if the individual is a child (first period) or an adult (second period) | 0: childhood<br>1: adulthood |
| IQ | IQ describes the cognitive ability of the child. Its value is fixed and determined by the IQ of the two parents and a random term. | $\mathcal{N}$(100,15). In the education production function $IQ/100$ is used. |
| Woman | Dummy variable for woman | 0: male<br>1: female |
| Education | Education level of the individual | Continuous on [0,1], where 0 refers to no education and 1 to highest possible education |
| Wage | Wage earned by the individual | Positive and continuous, with $E[wage \| educ. = 0] = 1$ |

can be interpreted as a multiple of the base income.

### *Families*

Families are composed of two parents (age=1) and an endogenous number of children (age=0). In addition to the state variables of each individual, the family is characterized by the variables displayed in Table S1.2: All the scales of the family state variables are directly linked to the individual wages. The utility has no fixed scale and no normalization was used in the model. However, a posterior normalization by putting the average utility of the baseline model was sometimes used in the discussion of the results.

Table S1.2: State variables of families

| **Variable** | **Description** | **Scale** |
|---|---|---|
| Family income | Sum of father's and mother's income | $\mathbb{R}_+$ |
| Tax | Tax payment | $\mathbb{R}_+$ |
| Subsidy | Subsidies received by the government | $\mathbb{R}_+$ |
| Invest | Investment in the education of children | $\mathbb{R}_+$ |
| Consumption | Consumption of the family | $\mathbb{R}_+$ |
| Utility | Realized utility (once children are educated) | $\mathbb{R}_+$ |

### *Government*

The government is only required when analyzing the policy interventions, otherwise the state variables presented in Table S1.3 become irrelevant.

All state variables are describing how the (conditional) cash transfer program is implemented. The tax rate is the only endogenously determined value of the government state variables. All other variables are exogenously set.

Table S1.3: State variables of the government

| Variable | Description | Scale |
|---|---|---|
| Subsidy type | Type of the government program according to table 4 | Categorical |
| Subsidy rate | Indicator $\eta$ for the size of the subsidies | $\mathbb{R}_+$ |
| Tax rate | Proportional tax rate for all families | Continuous on [0,1] |
| Eligibility threshold | Threshold dividing the population in those eligible to receive subsidies or not | Continuous on [0,1] if education is the criterion and $\mathbb{R}_+$ if income is the criterion |

### S1.1.3 Process overview and scheduling

In each period several steps take place and once all steps are completed, the next period begins. Table S1.4 displays the sequence of all steps taking place in each period. Note that the order of processes is not in all cases crucial. For instance whether individuals first search a partner and then get the wage is irrelevant because the wage is not used in the matching process. In contrast, in other cases the order is obviously crucial. For instance, couples can pay taxes and get children only after having met and after having received the wages. A more detailed description of each step is provided in the *Details* section (S1.3) of this ODD protocol.

Table S1.4: Sequence of steps taking place in each period

| # | Step | Entity | Action |
|---|---|---|---|
| 1 | 1 | Individuals | Children become adults and the adults from the previous period leave the model |
| 2 | 1 | Individuals | Partner search: Individual search a parter and in case of finding one, a new family is created. |
| 3 | 2 | Individuals | Each individual receives his/her wage |
| 4 | 2 | Government | Based on the subsidies paid in the previous and the income level of this period the government computes the tax rate |
| 5 | 2 | Family | Tax payment: families pay a proportional income tax |
| 6 | 3 | Family | Procreation: couples get children and transmit their ability through genes |
| 7 | 4 | Family | Education investment decision: families optimize their consumption and investment in education |
| 8 | 4 | Individuals | Children become educated according to the education investment |

**Notes:** The column step refers to the numbering of steps used in section 3

## S1.2 Design concepts

***Emergence***

In the model, several phenomena emerge while others are imposed by the model rules. The most important phenomena that emerge from the model are the intergenerational correlations in education and the education distribution. As a result of these emerging phenomena, the

resulting income and consumption distributions also emerge. On the other hand, the education dependent fertility rates and the spousal correlations in cognitive ability and education follow directly from the empirical rules. On the government side, the subsidy rate is exogenously imposed while the resulting tax rate emerges endogenously from the model.

***Objectives***

Families have the objective of optimizing their explicitly stated utility function, which considers both the consumption level and the expected education of the children. There is no explicitly modeled objective at the individual level.

***Prediction***

Families predict the expected education level of children by considering the assumed IQ level of the children (approximated by the average of the two parents) and their investment in education.

***Sensing***

When taking decision, the family knows the current subsidy and tax rate, their current income and the number of children. Moreover, the average parental IQ is used to estimate the IQ level of the children. They do not directly observe the IQ level of each child individually and therefore assume the same value for each child.

***Interaction***

The only active interaction between agents takes place on the marriage market, where individuals search a partner that corresponds to their criteria. To some extent the education investment decision can be seen as a passive interaction in the sense that the investment in one child depends on the presence of siblings. However, there is no active interaction or bargaining among siblings.

***Stochasticity***

There are three stochastic elements in the model. First, the marriage market is modeled by randomly choosing a partner among all the possible candidates satisfying the matching conditions. The order in which individuals search partners is also random. Second, the wage equation has a stochastic element, which is directly calibrated from the data. The third stochastic element is a random disturbance term in the education production function.

***Collectives***

Individuals are regrouped into families, which are always composed of two adults (mother and father) and an endogenously determined number of children.

***Observation***

Data is collected for posterior use at the individual, family and population level. In order to avoid

any impact of the initial conditions, the first 10 periods are never considered. For the model without government intervention only steady states are compared: the same periods using the same random seed but different settings (e.g. exclusion of some elements). For the evaluation of the policy intervention I focus on the comparison of the steady state several periods after the introduction of the policy measure. The outcomes under treatment are compared to the a simulation with the same random seeds but no treatment. Thus, all observed differences are exclusively due to the policy intervention. More details and examples of this data selection can be found in the S2 Appendix.

## S1.3 Details

### S1.3.1 Initialization

The model is initialized with a random population. Half of all individuals are women and the other half men. The cognitive ability (IQ) is drawn from a normal distribution $\mathcal{N}(100, 15)$ and the education level of the individual is derived using the education production function. As argument for the investment in education, a random value drawn from a uniform distribution between 0 and 3 is taken[2]. The model is insensitive to the exact choice of this distribution and converges within at most 5 periods to the same steady state. In the S2 Appendix, I present a typical example of the initialization of the model and discuss in detail how I deal with the first periods during the model initialization.

### S1.3.2 Input

The model does not require any type of input files, because the initial population is drawn from random distributions. The only exogenous input from the modeler concerns the government intervention. Besides the type of intervention, the modeler can also chose the moment when the intervention starts. In this study, I always introduce the government intervention in period 25. This allows the model to run on the steady state for several periods prior to the introduction of the government.

### S1.3.3 Submodels

In this section I will briefly outline the main mathematical implementations of the processes. This section complements the description in the main body of the paper.

***Partner search***

Based on empirical evidence, the partner search process is designed through conditions defining possible candidates. These conditions refer to the education and cognitive ability level of

[2]The value of 3 is chosen because it represents an upper level of investments when the model is stable. However, the initialization is not sensitive to this value. For instance, taking the value of 6 produces much larger average education levels in the first period but converges within 2 periods to the level based on the value of 3.

potential partner. The condition two individuals must satisfy is given by:

$$\delta_{IQ} \equiv |IQ_{woman} - IQ_{man}| \leq 30 \quad \wedge \quad \delta_{educ} \equiv |educ_{woman} - educ_{man}| \leq 1.5\sigma_{educ} \qquad (1)$$

All candidates $j$ satisfying this condition have equal probability of becoming the partner of individual $i$. Technically this is implemented using a double loop. The first loop goes through all individuals not yet in a couple. For each individual the program loops through the individuals of the opposite sex and selects the first individual satisfying the conditions. The order of individuals is random[3].

***Procreation***

The number of children a couple has follows a Poisson distribution

$$n_c(e_m) \sim \mathcal{P}(E[n_c|e_m]) \qquad (2)$$

where $E[n_c|e_m]$ is the expected number of children conditional on the education of the mother $e_m$. This value is based on an OLS regression using data from the 2010 Mexican Census. Table S1.5 displays this regression.

Table S1.5: Number of children as a function of maternal education

| Number of children (dep. var.) | Coef. | Std.Err |
|---|---|---|
| Mother's education | -4.3354*** | (0.0089) |
| Mother's education (squared) | 1.1941*** | (0.0081) |
| Constant | 5.0427*** | (0.0022) |
| N | 6544865 | |
| Adj. $R^2$ | .183 | |

*Source:* Author's calculation using the Mexican Census 2010.
**Notes:** Mother's education is normalized to the interval of 0 (no education) to 1 (highest education). Standard errors in parenthesis. Significance levels at 10% (*), 5% (**) and 1% (***).

I restrict the sample to women between 40 and 50 years old. Younger woman are excluded because they might still get more children, thus the observed number of children is probably not equal to the final number. The older women were excluded because they have substantially higher average number of children, which does no longer reflect the current situation in Mexico. Additionally I dropped extreme values of women with more than 25 children. The results are quite robust to small changes in these sample selection thresholds.

***Ability transmission through genes***

The ability transmission through genes is based on an equation that was calibrated using Mexican data. I make use of the Mexican Family Life Survey (MxFLS), where a short cognitive ability

[3] A previous version of the model first created the full set of possible candidates and then chose the partner randomly among these candidates. This provides exactly the same probability for each possible candidate as the currently implemented solution, but is much less computationally efficient.

test is included for both parents and their children.

Limiting the observations to all children where I have data on parental ability allows me to regress the ability score of the child on the same measure of the parents. For the purpose of readability, I scaled the ability measure to the IQ-scale[4], which is a normal distribution with mean 100 and standard deviation 15.

Table S1.6: Intergenerational transmission of ability

| Child's ability (Dep. var) | Coef. | Std.Err |
|---|---|---|
| Father's ability (IQ scale) | 0.2064*** | (0.0113) |
| Mother's ability (IQ scale) | 0.2854*** | (0.0113) |
| Constant | 51.8559*** | (1.2338) |
| N | 7936 | |
| Adj. $R^2$ | .168 | |

*Source:* Author's calculation, based on data from the Mexican Family Life Survey (MxFLS)
**Notes:** Standard errors in parenthesis. Significance levels at 10% (*), 5% (**) and 1% (***).

Table S1.6 reports the results of an OLS regression of ability on parental ability. The ability of the child is significantly influenced by the ability measures of both parents. However, the coefficient for the mother is significantly (F=17.05) higher than the one of the father. The unexplained part of children's ability follows a normal distribution $\mathcal{N}(0, 13.53)$. Thus, in the model the transmission is simulated as follows:

$$a_{\text{child}} = 51.8772 + 0.2059 \cdot a_{\text{father}} + 0.2857 \cdot a_{\text{mother}} + \mathcal{N}(0, 13.53) \qquad (3)$$

where $a$ denotes the cognitive ability (IQ). This implementation generates an IQ correlation with the father of approximately 0.33, which is very close to the correlation of 0.346 found by Björklund et al. (2010) and 0.38 computed by Black et al. (2009). The aim of this estimation is not to distinguish the biological transmission from the environmental effects, as it is controversially discussed in the literature. The unique aim is to reproduce the total intergenerational link in ability without going into the details of a decomposition in different sub-processes.

***Education production function***

The education of a child is a function of the cognitive ability of the child, the investment in the education and a stochastic element:

$$e(a, i) = \gamma_1 i^{\gamma_2} a^{\gamma_3} \epsilon_e \qquad (4)$$

This equation was indirectly calibrated through the model as described in the main body of the

[4]Note that the ability measure of the MxFLS is not a complete IQ test. Therefore it should only be considered as a proxy measure for the IQ.

paper (section 3.6). The calibrated values in the baseline model are:

$$e(a,i) = 0.4 i^{0.8} a^{1.0} \epsilon_e \qquad \text{with } \epsilon_e \sim \mathcal{N}(1, 0.2) \tag{5}$$

### *Wage equation*

The wage equation is based on a Mincer equation and can be written as follows:

$$w = \exp(\beta_0 + \beta_1 e + \epsilon_w) \tag{6}$$

In this study, I normalize the expected income without education to 1. This implies that $\beta_0 = 0$ and allows us to interpret $w$ as a multiple of the base wage. The calibrated version of the equation has $\beta_1 = 1.607$ and $\epsilon_w \sim \mathcal{N}(0, 0.756)$.

### *Investment decision*

The investment decision determining the investment $i$ in the education of each child is based on maximizing the following utility function:

$$U(i) = [W - i \times n_c - T(W) + S(i, n_c, W)]^{\alpha} \left[E[e_c(a,i)]^{n_c}\right]^{1-\alpha} \tag{7}$$

where $W$ is the income of the family, $i$ the investment in each child, $n_c$ the number of children, $T(W)$ the taxes to pay, $S(i, n_c, W)$ the expected education subsidies and $\alpha$ a parameter of the relative importance of education and consumption respectively. This parameter $\alpha$ was indirectly calibrated through the model as described in section 3.6 and takes the value of 0.5 in the baseline model.

### *Tax and subsidy rates*

The model uses a proportional income tax with the same tax rate for all individuals:

$$T(W) = \tau W \tag{8}$$

The tax rate is obtained by dividing the amount of subsidies paid in the previous period over the total income of the current period: $\tau = \frac{\sum S_{t-1}}{\sum W_t}$. This implementation ensures a balanced government budget and implies that every generation pays as adult what they received in childhood.

In this study different subsidy schemes are introduced. They are described in the main text and summarized in Table 4.

## References

**Björklund, Anders, Karin Hederos Eriksson, and Markus Jäntti**, "IQ and Family Background: Are Associations Strong or Weak?," *The B.E. Journal of Economic Analysis & Policy*, 2010, *10* (1).

**Black, Sandra E., Paul J. Devereux, and Kjell G. Salvances**, "Like Father, Like Son? A Note on the Intergenerational Transmission of IQ Scores," *Economic Letters*, 2009, *105* (1), pp. 138–140.

**Grimm, Volker, Uta Berger, Donald L. DeAngelis, J. Gary Polhill, Jarl Giske, and Steven F. Railsback**, "The ODD protocol: A review and first update," *Ecological Modelling*, 2010, *221* (23), pp.2760–2768.

**_ , _ , Finn Bastiansen, Sigrunn Eliassen, Vincent Ginot, Jarl Giske, John Goss-Custard, Tamara Grand, Simone K. Heinz, Geir Huse, Andreas Huth, Jane U. Jepsen, Christian Jørgensen, Wolf M. Mooij, Birgit Müller, Guy Peer, Cyril Piou, Steven F. Railsback, Andrew M. Robbins, Martha M. Robbins, Eva Rossmanith, Nadja Rüger, Espen Strand, Sami Souissi, Richard A. Stillman, Rune Vabø, Ute Visser, and Donald L. DeAngelis**, "A standard protocol for describing individual-based and agent-based models," *Ecological Modelling*, 2006, *198* (1), pp. 115–126.
