# Supplementary material for: The effect of cash transfer programs on educational mobility
Source: PLoS One. 2018 Oct 19;13(10):e0205957. doi: 10.1371/journal.pone.0205957 (PMC6195296; doi:10.1371/journal.pone.0205957)
Supplement: S2 Appendix — This appendix provides an overview of the different simulations and how the data were obtained to generate the results presented in the study. (PDF) [file pone.0205957.s002.pdf]

S2 Overview of the simulations

Throughout the results section I present results based on different simulations. From each simulation I collect data at the individual, family and population level and use it afterward to present the results. The goal of this appendix is to provide a clear overview over the simulation strategy, the settings and the samples of data used in the result section. I will first discuss the parameters used in the simulations. Then I present the general strategy of the simulation by explaining how I deal with the initialization of the model and what periods I consider for the results. Finally, I provide an overview of the data samples used to obtain the different results in section 4.

S2.1 Overview of parameters

Table S2.1 lists the full set of parameters used in the baseline model. A detailed description on how these values were chosen can be found in section 3.

Table S2.1: Values of the parameters used in the baseline model

Parameter	Value	Explanation
**Utility function (Equation 8)**		
α	0.5	Parameter in the utility function: relative importance of current consumption and education of children
**Wage equation(Equation 4)**		
β_0	0	Wage equation: Base income without education (the base income is set to 1 in levels and consequently it is zero for log-wages)
β_1	1.607	Wage equation: Return to education
ϵ_w	$\mathcal{N}(0, 0.756)$	Random term in the wage equation
**Education production function (Equation 6)**		
γ_1	0.4	Education production function: Scaling factor for the wage
γ_2	0.8	Education production function: Parameter shaping investment to education
γ_3	1.0	Education production function: Parameter shaping ability to education
ϵ_e	$\mathcal{N}(1, 0.2)$	Random part in the education production function
**Assortative mating condition (Equation 1)**		
δ_{educ}	$1.5\sigma_e$	Maximal spouse difference in education (1.5 standard deviations)
δ_{IQ}	30	Maximal spouse difference in IQ (30 IQ points)
**Population size and government programs**		
$T(W)$	0	No taxes in the baseline model
$S(i, n, W)$	0	No subsidies in the baseline model
N	1000	Initial number of agents in the model

In the *S3 Appendix* I present sensitivity checks for some of the parameters, particularly those for which no empirical calibration was possible.

S2.2 Simulation strategy

In some agent-based models the results depend on the initial conditions and therefore it is important to test the sensitivity of the model to the initial conditions (Helbling, 2012). Fortunately, the model I present in this study is very insensitive to the initial conditions and quickly converges to a stable situation (steady state). A way to reduce the potential impact of initial conditions is to run the model for several periods and to use only the results of the model once it is stable Tesfatsion (2002, 2003). I follow this approach and exclude the first periods from the analysis. Let us have a look at how the model behaves at the very beginning of the simulation. Figure S2.1 displays the average education and the intergenerational correlation with the mother in function of the simulation tick (period) in the absence of any government intervention. In

Figure S2.1: Convergence after initialization

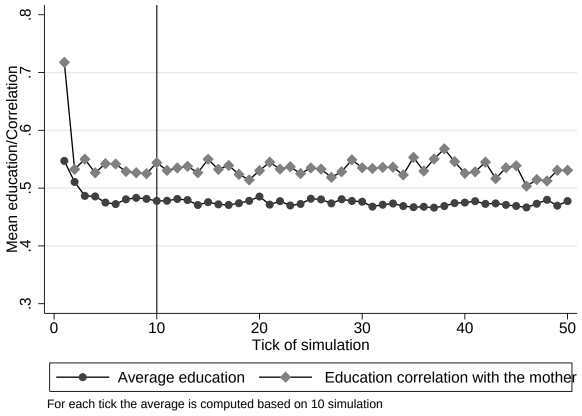

this example the initialization values are substantially higher than the steady state values. I chose this example because it clearly shows that even if the starting values are quite different, the model quickly converges to a stable situation (steady state). The level of this steady state is independent of the initial conditions. To ensure that all results are completely independent of the initial conditions, I never use the first 10 periods of the simulation in the results. The vertical line in the graphs depicts this period and we can see that at that point of time the model is already stable for several periods[1]. Hence, if the objective is to compare two steady states, we can simply simulate the two settings with the same random seed and compare the values at the steady state. By repeating this procedure for different random seeds we obtain a large number of observations at the population level. For instance, if we use 10 different random seeds and compare periods 11 to 50 we will have $10 \times 40 = 400$ data points at the population level[2].
In case of analyzing policy interventions the strategy is slightly more complicated because more stages must be considered. If we would introduce the policy from the beginning there might

[1] I tested the steady state conditions using a regression with a linear trend and with linear splines. In most cases the steady state was already achieved after 3 to 5 periods. However, in order to avoid any risk of slower convergence, I always exclude the first 10 periods from the analysis.

[2] This example corresponds to the strategy used to create Table 3 for instance.

be a non-equal reaction to the initial conditions under policy intervention as compared to a government free simulation. In such as case the starting point under the policy intervention would be different than in the baseline and we would confound the effects of the policy measure with this difference. To avoid this, I first simulate the model without policy interventions until the model is stable and then I introduce the policy. Figure S2.2 depicts this using the example of the intergenerational correlation with the father.

Figure S2.2: Different stages of the simulation

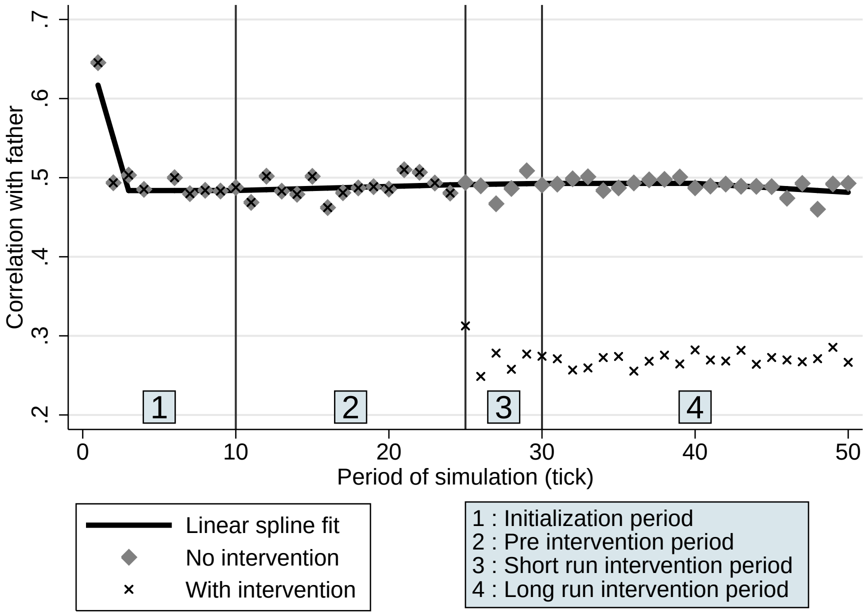

The gray markers refer to the simulation without any policy intervention, while in case of the black markers the policy intervention starts at period 25. Both simulations are based on the exactly same random seed. The solid line refers to a linear spline regression allowing us to analyze if there is a significant trend in the data at each stage of the simulation. In this case, the only slope that is significantly different from zero is the first spline for periods one to three. For the remaining splines we cannot reject the hypothesis of a steady state as the coefficients are not significantly different from zero. Now, let us have a closer look at the four stages indicated in the graph and divided by vertical lines.

The whole simulation includes 50 periods, which are divided into four stages. The first stage is the initialization period of 10 ticks. The data from this first stage are never used for the presentation of results in this study to avoid any kind of initialization effects.

The second stage is a pre-intervention steady state phase. The third stage starts in period 25 when the policy intervention is introduced. The policy intervention period is divided into two sub-periods: the short and the long run effects. We can see that once the policy measure is introduced the model almost immediately jumps to the new steady state. Even though the

effects are almost instantaneous, it can be interesting to analyze in detail this short run period[3] In contrast, if we want to focus on the steady state comparisons, I start using the data-points only five periods after the policy intervention was introduced. This rather short period can be justified by the almost immediate jump to the new steady state we can observe in Figure S2.2. Figure 3 in the main body of the article is an example for such an analysis.

I will now present an overview of the different simulation settings used to produce the tables and figures in the result section of this study.

S2.3 Sample definitions

Table S2.2 provides an overview of the samples used to produce the different figures and tables in section 4. The column *Runs per setting* refers to the number of different random sees used and column *Used periods per run* shows how many periods (ticks) were taken from each of them. The *number of data points* refers to the number of data point used for each statistic. As statistic are considered for instance one correlation, one point in a scatter plot or one curve when using a non-parametric regression. The column *Steady state comparison* indicates whether the situations were compared at the short run (stage 3 in Figure S2.2) or at the steady state (stage 4 in Figure S2.2).

Table S2.2: Overview of samples used in the results section

Output	Runs per setting	Used periods per run	Number of data-points	Steady state comparison	Remarks
Table 3	10	40	400	yes	
Figure 2	4	20	80	yes	
Figure 3	10	21	≈ 210K	yes	The number of data-points refers to each line
Figure 4	5	20	100	yes	
Sensitivity analysis	4	20	≈ 80K	yes	The number of data-points refers to each line

The baseline results where no policy intervention is introduced is based on 10 different random seeds and from each of them I consider 40 periods. Hence, each statistic presented in Table 3 is the average out of 400 population statistics, each based on several thousands of individuals. In addition to the reported results in this study I also varied the number of data points considered to see whether this affects the results. In no case changing the number of data points had an impact on the results.

References

Helbling, Dirk, "Agent-Based Modeling," in Dirk Helbling, ed., *Social Self-Organization: Agent-Based Simulations and Experiments to Study Emergent Social Behavior*, Springer-

[3]For reasons of space this is not discussed in this article. Interested readers can find such an analysis in Wendelspiess Chávez Juárez (2014).

Verlag Berlin Heidelberg, 2012.

Tesfatsion, Leigh, “Agent-Based Computational Economics: Growing Economies From the Bottom Up,” *Artificial Life*, 2002, *8* (1), pp. 55–82.

_ , “Agent-based computational economics: modeling economies as complex adaptive systems,” *Information Sciences*, 2003, *149*, pp. 263–269.

Wendelspiess Chávez Juárez, Florian, “Three essays on inequality of opportunity and social mobility,” PhD Thesis, University of Geneva. Available at http://archive-ouverte.unige.ch/unige:40023 2014.
