# Supplementary material for: The effect of cash transfer programs on educational mobility
Source: PLoS One. 2018 Oct 19;13(10):e0205957. doi: 10.1371/journal.pone.0205957 (PMC6195296; doi:10.1371/journal.pone.0205957)
Supplement: S3 Appendix — This appendix provides a series of sensitivity checks of key parameters of the model. (PDF) [file pone.0205957.s003.pdf]

S3 Sensitivity checks

In this appendix I first present sensitivity checks for different parameters and at the end of the appendix I discuss them in general.

S3.1 Sensitivity to parameters

With the complexity of the model, the number possible sensitivity checks that can be done increase sharply. In this section I present some sensitivity checks by changing main parameters, especially those I was not able to estimate directly. Besides the possible values to include in the sensitivity checks, there are also different ways to present them. I present here the graphs showing the relationship between education and the parental education as I consider them to be the most informative. In addition to the purely graphical approach, I also report in each graph the intergenerational correlation with the mother for the case of no intervention. Table S3.1 displays the parameters I am changing in the sensitivity checks.

Table S3.1: Sensitivity checks

Equation	Para-meter	Value in baseline model	Values in sensitivity check
Wage equation	β_1	1.607	1.5, 1.607, 1.7
	ϵ_w	$\mathcal{N}(0, 0.756)$	$\mathcal{N}(0, 0.70)$, $\mathcal{N}(0, 0.756)$, $\mathcal{N}(0, 0.80)$
Educ equation	γ_2	0.80	0.75, 0.80, 0.85
	γ_3	1.00	0.90, 1.00, 1.10
Utility	α	0.50	0.40, 0.45, 0.50, 0.55, 0.60

The presented sensitivity checks are all performed using the government intervention of Type EI. Under the Type EI scheme eligible families are chosen based on parental education whereas the amount of subsidies is based on income.

S3.1.1 Utility function

The main parameter of the utility function that might change the analysis is α, the relative importance of current consumption compared to the education of children. In the baseline model I assume a value of 0.5. Here I compare this baseline value to values from 0.40 (giving more weight to the education of children) to 0.60 (more weight on current consumption). Figure S3.1 displays the graphs for these sensitivity checks and the baseline model.

The most important conclusion we can draw from these graphs is that the general pattern does not change with α. Under the program the increase in average education for the net receivers is large and easily offsets the loss at the top. The average education of children with poorly educated parents is slightly higher when we use a lower value of α. The reason for this is that more weight is put on education and therefore families tend to invest more in education. At

Figure S3.1: Sensitivity checks for α

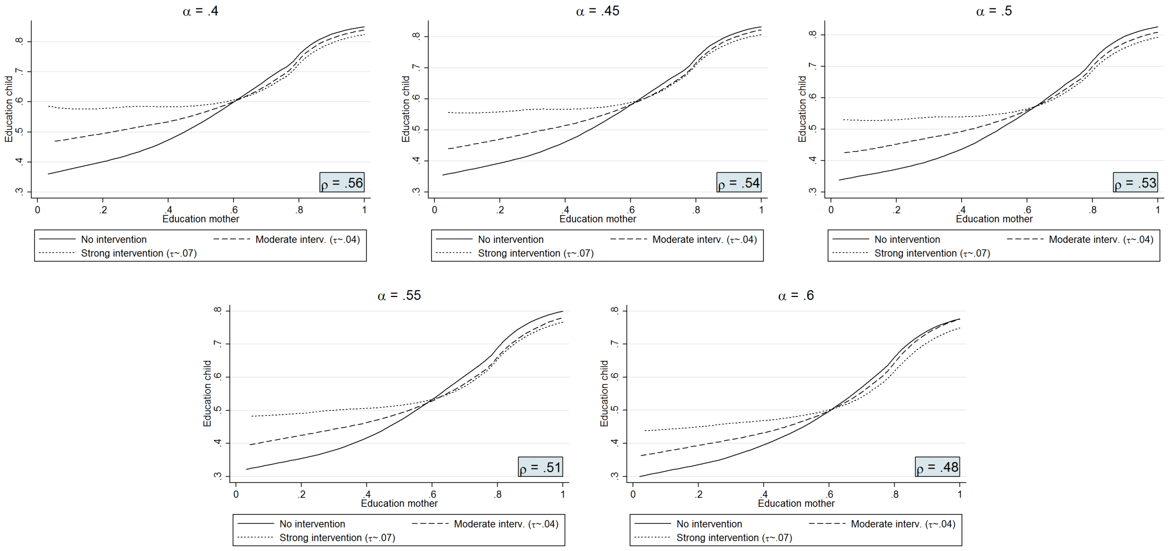

Notes: Each curve is based on approximately 80,000 individuals. These individuals were obtained from 20 periods for 4 different random seeds.

the top this does not change the average education because they were already at very high levels of investments in education where the marginal effect becomes smaller. The effect of the program becomes also more important when α is lower, which is in line with the previous point. When people care more about education, they react more to subsidies. The intergenerational correlation goes somewhat down when increasing the value of α.

S3.1.2 Education production function

For the education production function three parameters are of interest for the sensitivity check. γ_2 is the sensitivity of education to ability and γ_3 shapes the investment to education. The variance of the disturbance term is a third parameter that could potentially affect the results. Finally, the last parameter γ_1 is not included in the sensitivity check as its only purpose is to keep the model stationary over several generations. Figure S3.2 presents the sensitivity checks for γ_2 and γ_3, where the graph in the middle corresponds to the baseline, γ_2 increases from top to bottom and γ_3 from left to right. Again, the quality of the results does not change with the two parameters γ_2 and γ_3. In this case, the intergenerational correlations remain very stable across the different settings. When looking at the effect of γ_3 we cannot observe any substantial difference. By increasing γ_2 we observe a slightly larger effect of the government program. This is due to the fact that by increasing γ_2 the link between investment and education is strengthened and therefore a government investment targeting the investment in education becomes more effective.

Additionally to the main parameter of the education production function I present in Figure S3.3 sensitivity checks on the variance of the disturbance term.

Figure S3.2: Sensitivity checks for the education equation

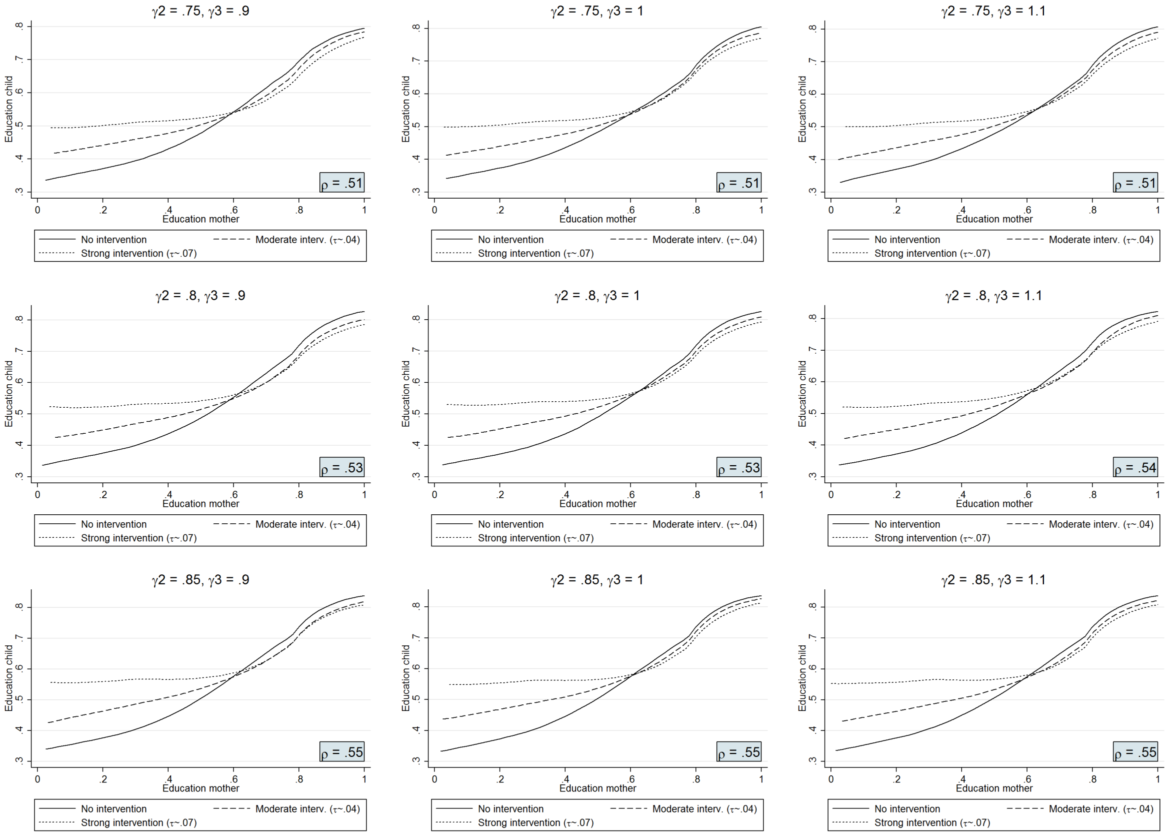

Notes: Each curve is based on approximately 80,000 individuals. These individuals were obtained from 20 periods for 4 different random seeds.

Figure S3.3: Sensitivity checks for the education equation

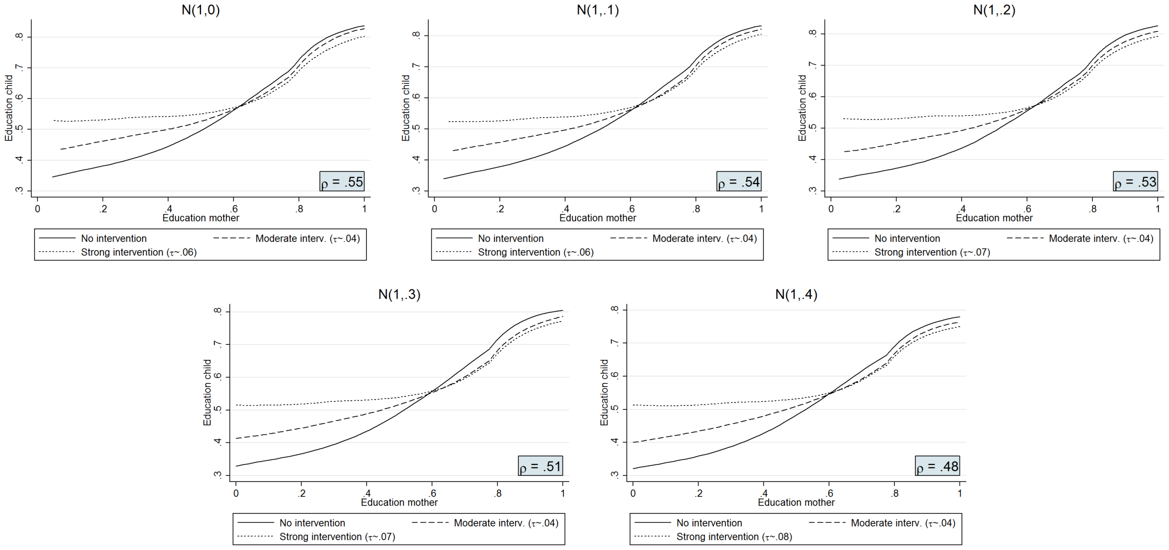

Notes: Each curve is based on approximately 80,000 individuals. These individuals were obtained from 20 periods for 4 different random seeds.

In general the results are very robust with respect to this parameter. Only in the case of a very large disturbance variance the intergenerational correlation decreases substantially. The reason

for this is that by increasing the variance the link between investment and the outcome become less clear and therefore the intergenerational correlations are reduced even if the inequalities in the investment remains the same.

S3.1.3 Wage equation

For the wage equation I show the sensitivity check for two parameters of interest. I let both the return to education β_1 and the variance of the stochastic element vary. Figure S3.4 displays the graphs for different combinations of these two parameters. From left to right the variance of the stochastic element increases and from top to bottom the return to education increases. The graph in the middle corresponds to the baseline case. Interestingly the results are extremely robust

Figure S3.4: Sensitivity checks for the wage equation

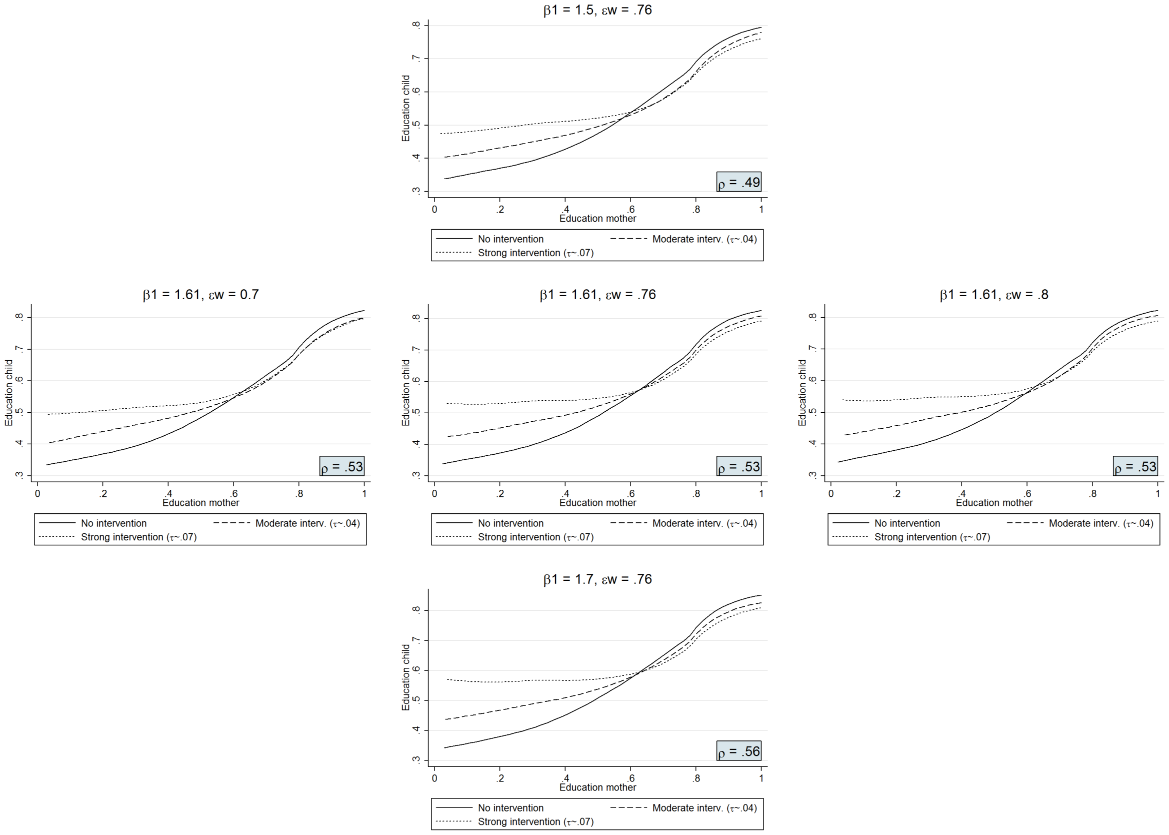

Notes: Each curve is based on approximately 80,000 individuals. These individuals were obtained from 20 periods for 4 different random seeds.

to the changes in the variance of the disturbance term. In contrast, the return to education has some effect on the intergenerational correlations. The higher the return to education is, the higher is the intergenerational correlation. The logic behind this result is that the link between parental education and wages become stronger and therefore also the intergenerational links. The differences between the poor and the rich in terms of wages is increased and therefore also the inequality in education.

S3.2 General discussion on sensitivity checks

As a general conclusion on the sensitivity checks we can probably say that the qualitative results presented in section 4 and especially in section 4.2 are robust to changes in the parameters. The exact quantitative results, however, depend somewhat on the calibration. This need not be a problem of the model, as it reflects that the effects of a policy measure depend on the context. It is reasonable to believe that in a different context (e.g. using data from another country), the parameters would be slightly different and the quantitative results as a consequence too.
